# New record and redescription of *Calanopia thompsoni* A. Scott, 1909 (Copepoda, Calanoida, Pontellidae) from the Red Sea, with notes on the taxonomic status of *C. parathompsoni* Gaudy, 1969 and a key to species

**DOI:** 10.3897/zookeys.552.6180

**Published:** 2016-01-13

**Authors:** Ali M. Al-Aidaroos, Adnan J. Salama, Mohsen M. El-Sherbiny

**Affiliations:** 1 Department of Marine Biology, King Abdulaziz University, Jeddah 21589, Saudi Arabia; 2 Department of Marine Sciences, Suez Canal University, Ismailia 41522, Egypt

**Keywords:** New record, copepods, Calanopia thompsoni, Pontellidae, Red Sea

## Abstract

During a plankton sampling programme around Al-Wajh area, Saudi Arabian coast of the northern Red Sea, a copepod *Calanopia thompsoni* A. Scott, 1909 (Calanoida: Pontellidae) was reported for the first time in the Red Sea. Both sexes are fully redescribed and compared to previous descriptions as well as the closely related species, *Calanopia parathompsoni*. The zoogeographical distribution of the species confirms that it is of Indo-Pacific origin. A dichotomous key for the identification of males and females of the species of *Calanopia* is included.

## Introduction

Recent studies of the neritic and coastal waters of the Red Sea have revealed an assemblage of calanoid copepods including several species new to science (Ohtsuka et al. 2000, El-Sherbiny and Ueda 2008a, 2010, El-Sherbiny 2011, El-Sherbiny and Al-Aidaroos 2015), in addition to several new records (El-Sherbiny and Ueda 2008b, El-Sherbiny 2009, El-Sherbiny and Al-Aidaroos 2013, 2014). The genus *Calanopia* accommodates 17 nominal species (Razouls et al. 2015). Most members of this genus (13 species) are Indo-Pacific species, namely *Calanopia aurivilli* Cleve, 1901, *Calanopia australica* Bayly & Greenwood, 1966, *Calanopia asymmetrica* Mulyadi & Ueda, 1996, *Calanopia elliptica* Dana, 1852, *Calanopia herdmani* A. Scott, 1909, *Calanopia media* Gurney, 1927, *Calanopia minor* A. Scott, 1902, *Calanopia parathompsoni* Gaudy, 1969, *Calanopia sarsi* Wilson, 1950, *Calanopia thompsoni* A. Scott, 1909, *Calanopia sewelli* Jones & Park, 1967, *Calanopia seymouri* Pillai, 1969, and *Calanopia kideysi* Ünal & Shmeleva, 2002 (Silas and Pillai 1973, Mulyadi and Ueda 1996). Of the remaining species, *Calanopia biloba* Bowman, 1957 and *Calanopia americana* Dahl, 1894 are known from the Atlantic Ocean (Bowman 1957), while *Calanopia levantina* Uysal & Shmeleva, 2004 and *Calanopia metu* Uysal & Shmeleva, 2004 were identified from Mediterranean Sea. In the Red Sea, only four species of the genus *Calanopia* have been recorded namely: *Calanopia elliptica* by Giesbrecht (1896), *Calanopia minor* by A. Scott (1902), *Calanopia media* by Pesta (1941) and *Calanopia kideysi* by Ünal and Shmeleva (2002).

The general morphological characteristics of *Calanopia* species collected from the Red Sea were close to those of *Calanopia thompsoni* described from Bay of Kankamaraan, south coast of Kangeang Island by A. Scott (1909) and *Calanopia parathompsoni* collected from neritic waters of Madagascar by Gaudy (1969). Since the original description of *Calanopia thompsoni* is incomplete and the literature from different areas notes morphological variability, the present paper provides a full redescription especially of the mouthparts which have never been described and figured. Also, this paper records the first occurrence of *Calanopia thompsoni* in the Red Sea and discusses its relationship to *Calanopia parathompsoni*.

## Material and methods

Within the plankton sampling framework of a study of the reproductive cycle and larval stages of the spiny lobster, *Panulirus penicillatus* (Decapoda: Palinuridae) in Al-Wajh waters (26°11.855'N, 36°25.58'E) off the east coast of Saudi Arabian Red Sea, an unrecorded species of *Calanopia* was collected. Specimens were sampled using a 50-cm diameter plankton net (500 µm mesh size) towed near the surface for 15 minutes at a speed of about 2 knots. Immediately after sampling, samples were fixed in a 4% formalin-seawater solution and later *Calanopia* specimens were sorted and kept in 70% alcohol. For microscopic examination, dissections were made in polyvinyl lactophenol using bright-field and differential interference microscopes (Nikon DM 6000). Drawings were made with a camera lucida attached to the microscope. Terminology follows Huys and Boxshall (1991). For scanning electron microscopy, specimens were washed in filtered seawater, clean distilled water, and dehydrated through an 30-100% ethanol series and subsequently, critical-point-dried. The specimens were mounted on a stub, coated with gold palladium, and observed with a SEM Hitachi S-3500N.

## Results

### Description Order Calanoida G. O. Sars, 1903 Family Pontellidae Dana, 1853 Genus *Calanopia* Dana, 1853

#### Calanopia thompsoni

Taxon classificationAnimaliaCalanoidaPontellidae

A. Scott, 1909

**Figure 1.**
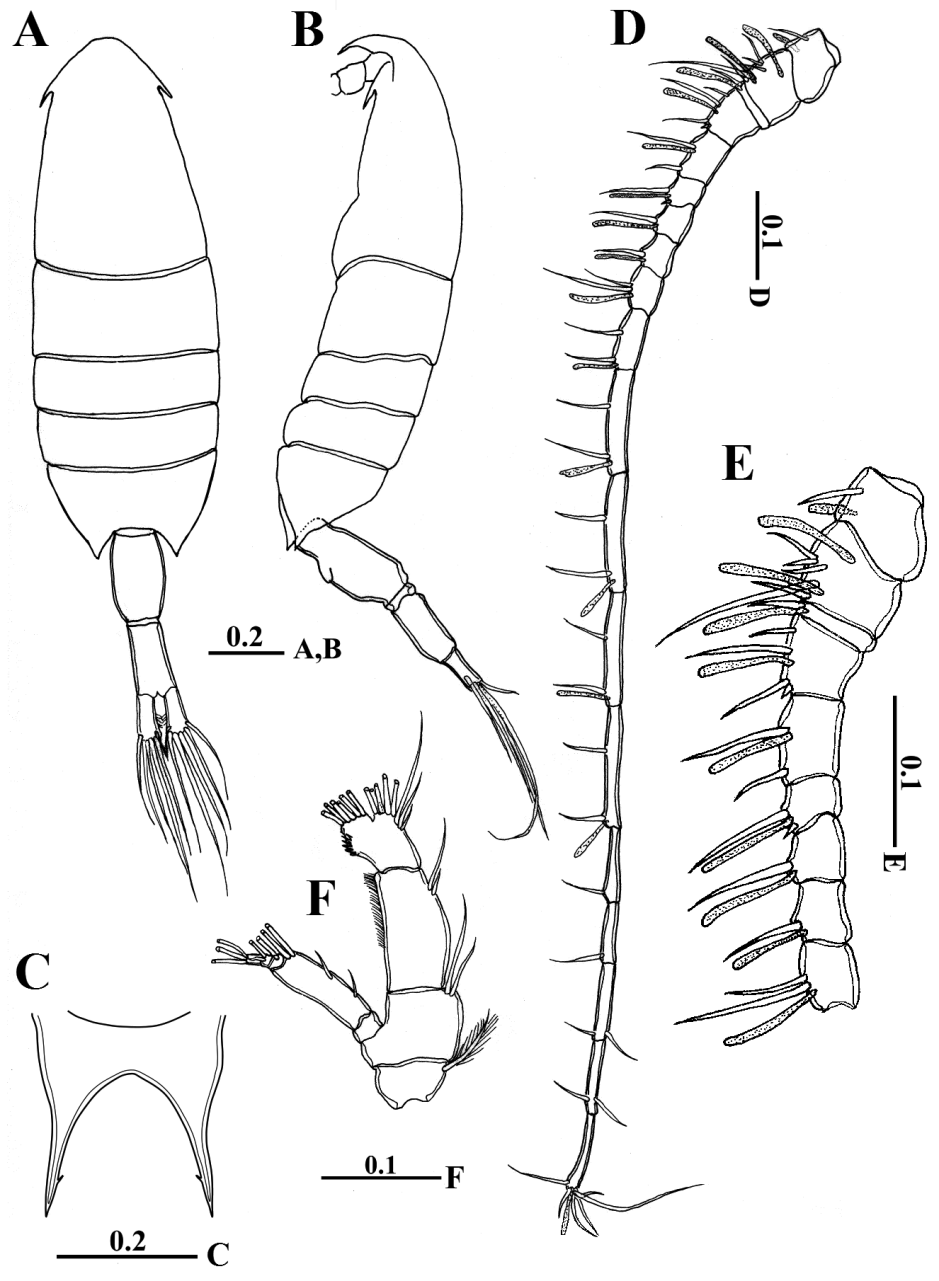
*Calanopia thompsoni* female from the Red Sea. **A** habitus, dorsal view **B** habitus, lateral view **C** rostrum, frontal view **D** antennule **E** enlarged proximal part of antennule **F** antenna. Scale bars in mm.

**Figure 2.**
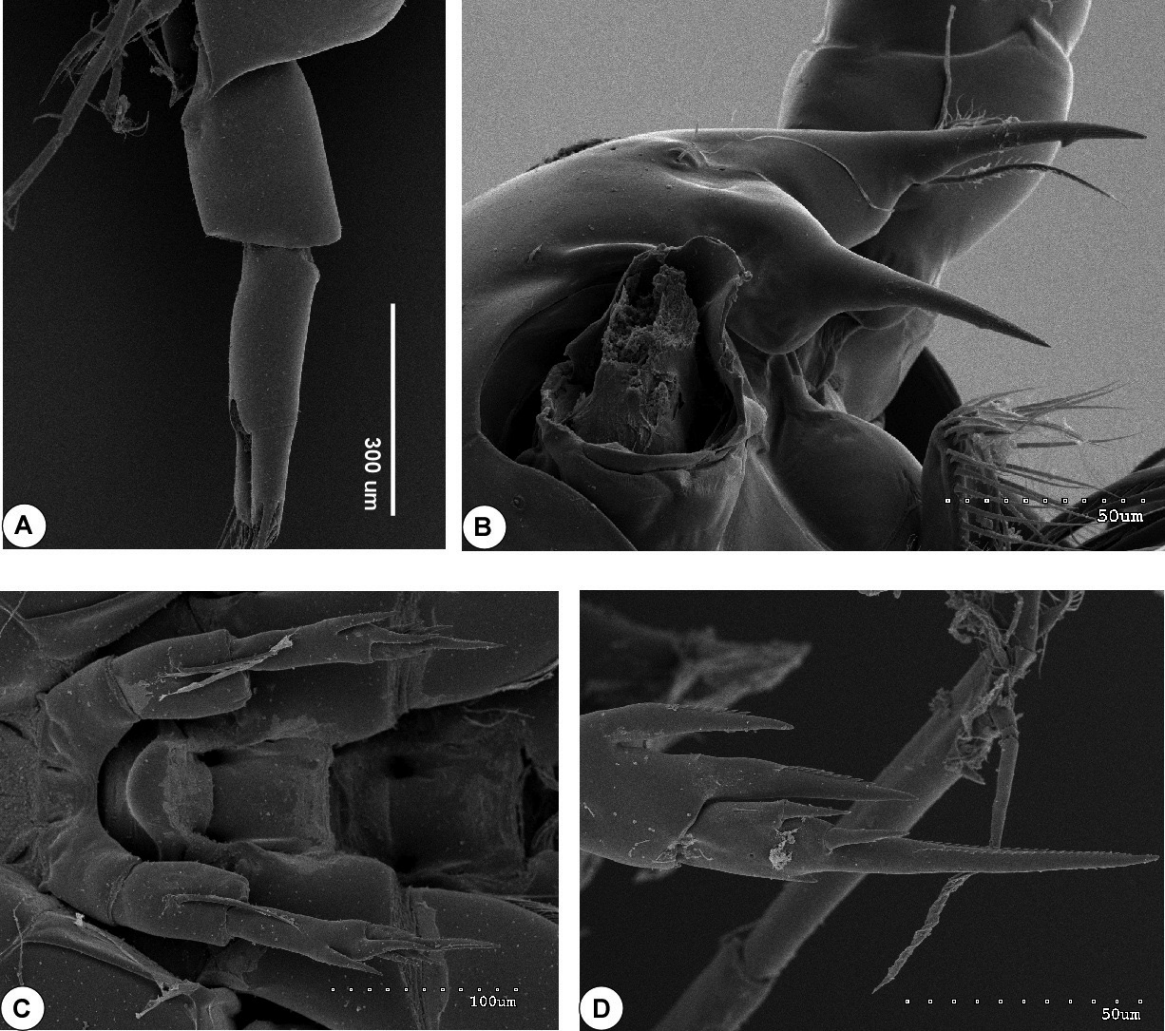
SEM micrograph of *Calanopia thompsoni* from the Red Sea. **A** female abdomen, lateral view **B** rostrum, lateral view **C** leg 5, ventral view **D** enlarged distal part of female leg 5.

**Figure 3.**
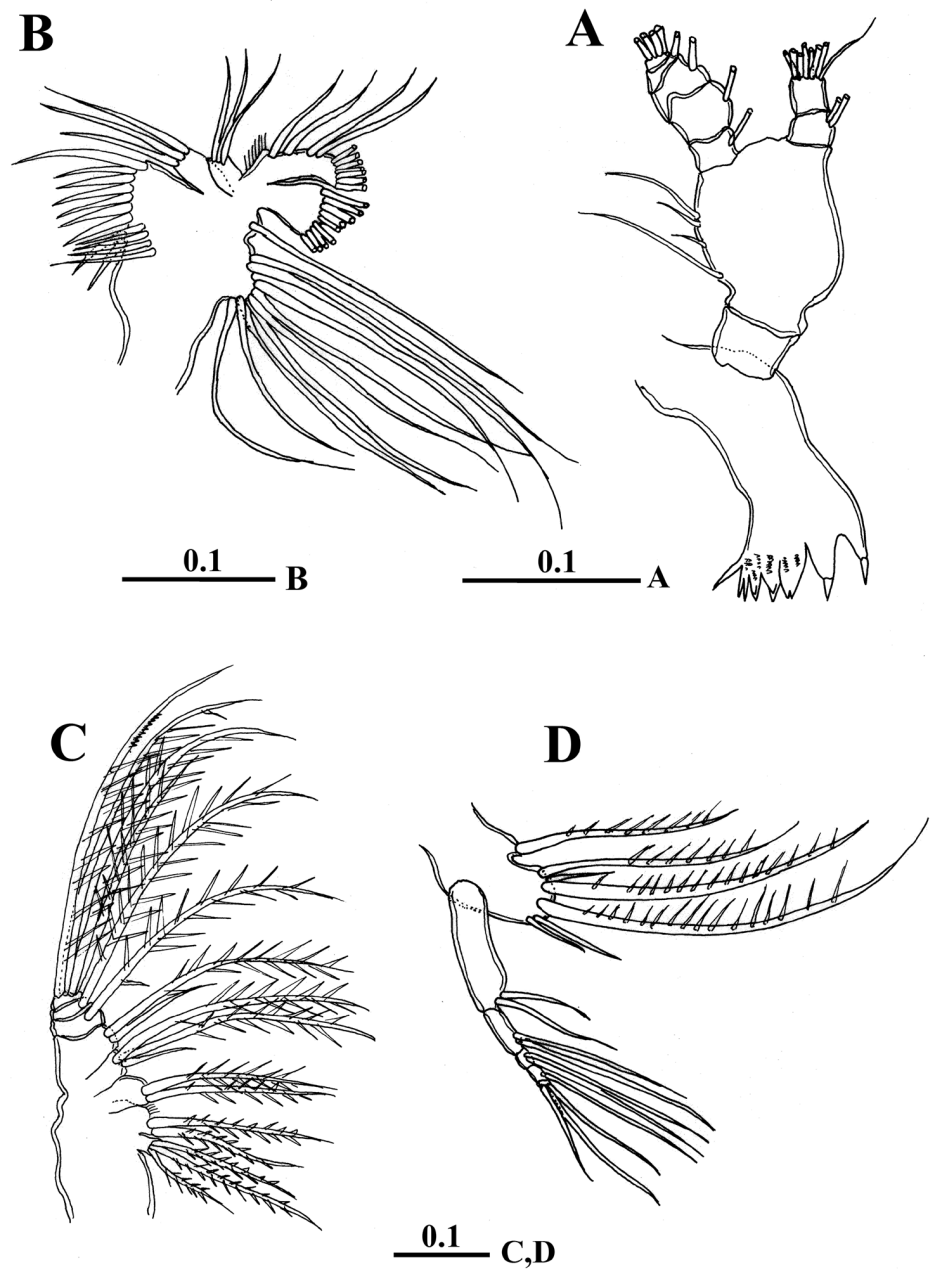
*Calanopia thompsoni* female from the Red Sea. **A** mandible **B** maxillule **C** maxilla **D** maxilliped. Scale bars in mm.

**Figure 4.**
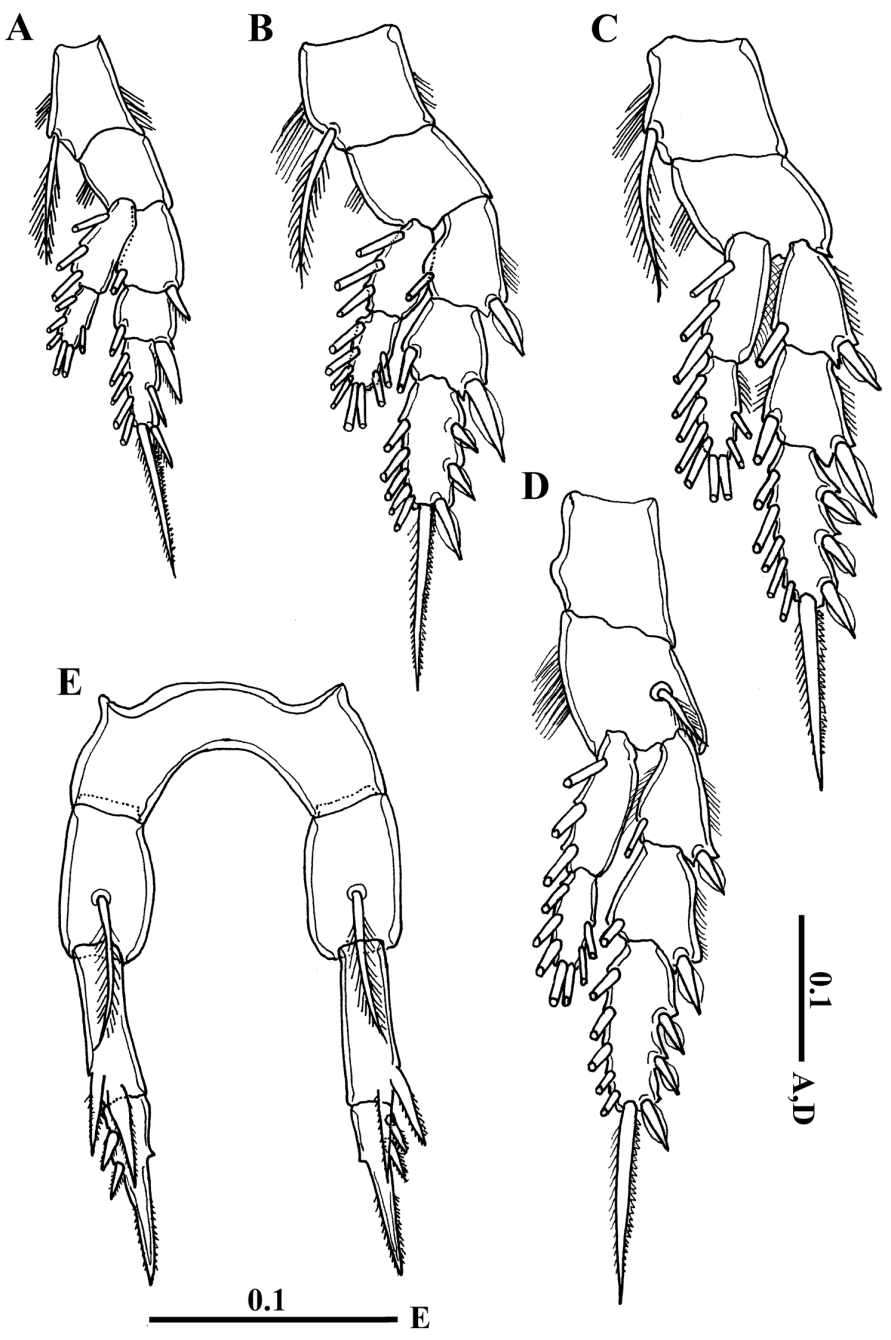
*Calanopia thompsoni* female from the Red Sea. **A** leg 1 **B** leg 2 **C** leg 3 **D** leg 4 **E** leg 5. Scale bars in mm.

**Figure 5.**
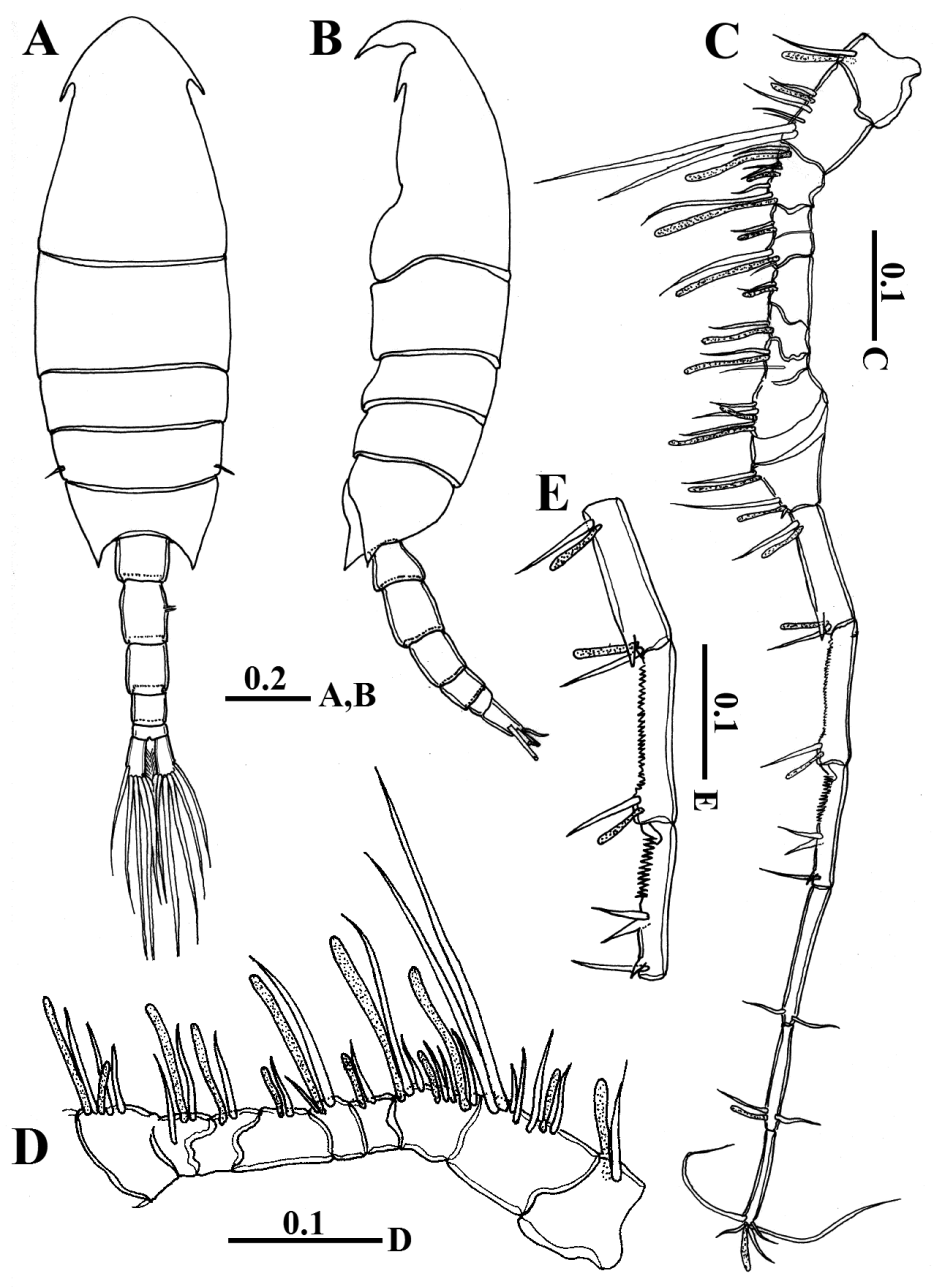
*Calanopia thompsoni* male from the Red Sea. **A** habitus, dorsal view **B** habitus, lateral view **C** right antennule **D** enlarged proximal part of right antennule **E** antennule segments 12–14. Scale bars in mm.

**Figure 6.**
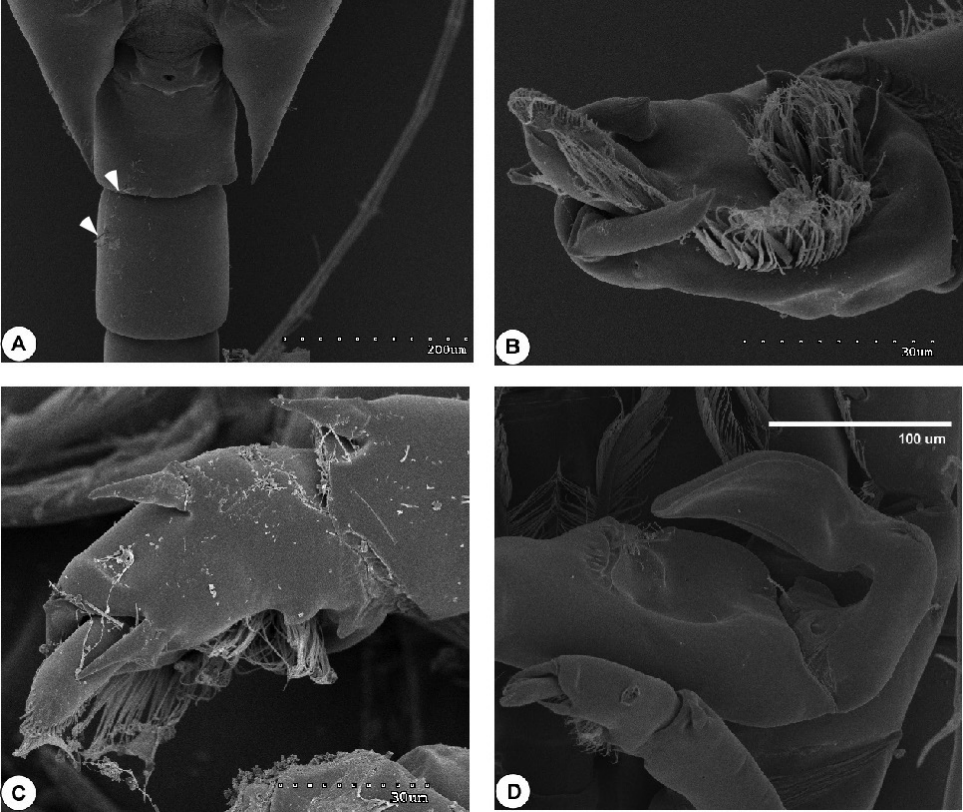
SEM micrograph of *Calanopia thompsoni* from the Red Sea. **A** first and second male urosomite (spinules indicated by arrows), ventral view **B** distal segment of male left leg 5, ventral view **C** distal segment of male left leg 5, dorso-lateral view **D** exopod of male right leg 5.

**Figure 7.**
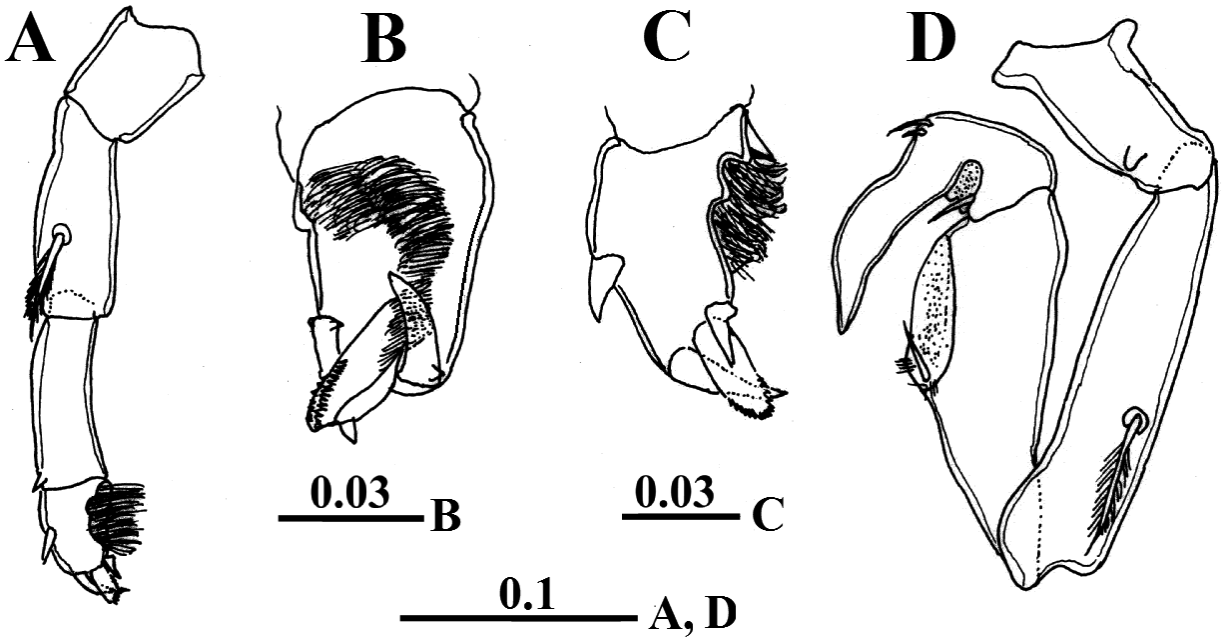
*Calanopia thompsoni* male from the Red Sea. **A** left leg 5 **B** distal segment of left leg 5, ventral view **C** distal segment of left leg 5, dorso-lateral view **D** right leg 5. Scale bars in mm.

Figs 1 , 2 , 3 , 4 , 5 , 6 , 7

##### Material examined.

Twelve adult females and ten adult males collected at Al-Wajh waters of the east coast of Saudi Arabian Red Sea.

##### Body length.

Female 1.92–1.98 mm (mean ± SD = 1.95 ± 0.02 mm, n = 12), male 1.79–1.83 mm (1.81 ± 0.01 mm, n = 10).

##### Female.

Body robust (Fig. 1A), 1.94 mm in length. Prosome elliptical comprising cephalosome and four pedigerous somites, prosome approximately 2.5 times as long as urosome; cephalosome distinctly separated from first pediger with one median eye and lateral hooks; fourth and fifth pedigerous somites fused, symmetrical with posterolateral corners pointed in dorsal aspect reaching nearly one-third of way along genital compound somite (Fig. 2A). Rostrum bearing pair of pointed processes with very small medial subterminal notch (Figs 1B, C, 2B). Urosome (Figs 1A, B, 2A) of two free somites; genital compound somite symmetrical and ventral surface without any processes (Figs 1B, 2A). Second urosomite symmetrical and slightly shorter than genital compound somite. Caudal rami symmetrical and approximately 2.3 times as long as wide, each ramus carrying five plumose setae along distal margin and reduced seta (seta VII) located on dorsal surface near medial distal angle.

Antennules (Fig. 1D, E) 19-segmented, when extended reaching almost anterior border of second urosomite. Armature formula as follows: ancestral segment I (segment 1) = 1 setae + aesthetasc (ae), II-VI (2) = 5 + 2 ae, VII (3) = 1 + ae, VIII-X (4) = 4 (1 spiniform) + ae, XI-XII (5) = 2 + ae, XIII (6) = 2 (1 spiniform) +ae, XIV (7) = 1 + ae, XV (8) = 1 + ae, XVI (9) = 2 + ae, XVII (10) = 2 + ae, XVIII (11) = 2 + ae, XIX (12) = 2 + ae, XX (13) = 2 + ae, XXI (14) = 2 +ae, XXII (15) = 1, XXIII (16) = 1, XXIV (17) = 1 + 1, XXV (18) = 1 + 1, XXVI-XXVIII (19) = 6 + ae.

Antenna (Fig. 1F) biramous with short coxa bearing plumose seta at distomedial angle; basis with two subequal setae distomedially; exopod 5-segmented with setal formula of 0, 4, 1, 2, 3. Endopod 2-segmented, proximal segment with two unequal subterminal setae, distal segment bilobed, with medial (proximal) lobe bearing eight setae, and with lateral (distal) lobe crowned with six setae and transverse row of fine setules.

Mandibular gnathobase (Fig. 3A) carrying eight teeth on coxal cutting edge, third to seventh teeth ornamented with row of short spinules anterodistally at base. Palp biramous; basis with four unequal setae on medial margin. Exopod 5-segmented with setal formula of 1, 1, 1, 1, 4. Endopod 2-segmented, proximal segment with two setae at distomedial corner, distal segment with seven long and one short setae.

Maxillule (Fig. 3B) with praecoxal arthrite bearing nine marginal strong spines and four setae on posterior surface. Coxal epipodite with nine setae; coxal endite with three setae, basal exite with one seta. Proximal and distal basal endites with three and one setae, respectively. Exopod carrying a total of nine setae; endopod incorporated into basis with three setae laterally and seven setae terminally.

Maxilla (Fig. 3C) praecoxal and coxal endites carrying 3 and 2, 2, 3 bilaterally spinulate setae respectively; basal endite with two setae, one longer than other; endopod 3-segmented, carrying six bilaterally spinulate setae.

Maxilliped (Fig. 3D) syncoxal lobes with 1, 3, 3 setae on their medial margins. Basis carrying two setae distally; endopod 4-segmented with setal formula of 2, 2, 1, 3.

Swimming legs 1–4 (Fig. 4A–D) biramous, with 3-segmented exopods and 2-segmented endopods. On leg 1 to leg 3, coxa with one medial seta and patch of fine hairs. All lateral spines on exopods of legs 1-4 with serrated hyaline margins. Leg 5 (Figs 2C, 4E) symmetrical, basis with short seta posteriorly; exopod 2-segmented, first segment with two strong bilaterally serrated processes laterally (distal one longer and pointed slightly mediad). Second exopod segment nearly as long as first one, bearing two bilaterally serrated, lateral spines, one small medial process fused to segment and bilaterally serrated long, distal spine fused to segment (Figs 2C, D, 4E). Armature of legs as follows:

**Table:** Armature of legs

	Coxa	Basis	Exopod			Endopod	
			1	2	3	1	2
Leg 1	0-1	0-0	I-1	I-1	II, I, 4	0-3	1, 2, 3
Leg 2	0-1	0-0	I-1	I-1	III, I, 5	0-3	2, 2, 4
Leg 3	0-1	0-0	I-1	I-1	III, I, 5	0-3	2, 2, 4
Leg 4	0-0	1-0	I-1	I-1	III, I, 5	0-3	2, 2, 3

*Male*. Body (Fig. 5A, B) with plump prosome approximately 2.2 times as long as urosome comprising cephalosome and four pedigerous somites. Cephalosome distinctly separated from first pediger; fourth and fifth pedigerous fused and produced posterolaterally into symmetrical and slightly pointed corners reaching end of first urosomite (Figs 5A, 6A). Rostrum bearing pair of pointed processes directed posteroventrally (Fig. 5B). Urosome (Fig. 5A, B) comprising five free symmetrical urosomites, second urosomite longest; anal somite shorter preceding somite. Caudal rami symmetrical, 2.2 times longer than wide; caudal setae as in female. Some male specimens from Red Sea revealed the presence of one and/or two fine spinules, ventrally on the right side in the first and second urosomite respectively (Fig. 6A).

Right antennule (Fig. 5C–E) 17-segmented, geniculate between segments XX (13) and XXI-XXIII (14). Armature as follows: ancestral segment I (segment 1) = 1 setae + aesthetasc (ae), II-V (2) = 6 + ae, VI-VII (3) = 5 + 3 ae, VIII (4) = 1 + ae, IX (5) = 2 + ae, X (6) = 1 + ae, XI (7) = 1 + ae, XII (8) = 2 + ae, XIII-XIV (9) = 3 + 2 ae, XV (10) = naked, XVI-XVII (11) = 3 (1 spiniform) + 2 ae, XVIII-XIX (12) = 2+ process + 2 ae, XX (13) = 1+ ae, XXI-XXIII (14) = 2 + 2 processes , XXIV (15) = 1 + 1, XXV (16) = 1+ ae + 1, XXVI-XXVIII (17) = 5 + ae.

Left antennule, antenna, mouthparts and swimming legs 1-4 as in female. Leg 5 uniramous and asymmetrical. Left leg (Fig. 7A) with short coxa; basis 1.8 times longer than coxa with plumose seta located posteriorly near proximal end. Exopod 2-segmented, first (proximal) segment slightly shorter than basis with pointed attenuation near distolateral corner, second (distal) segment short, hirsute on posteromedial surface, with curved relatively long spine laterally, short spine with triangular base medially and one rounded and serrated process distally (Figs 6B, C, 7B, C). Right leg (Fig. 7D) longer than left, coxa with one blunt process on posterior surface distally; basis with plumose seta laterally. Exopod 2-segmented, forming a stout subchela, first exopodal segment without thumb and nearly 4 times as long as wide, distal part of subchela with elongate depression medially and one seta on proximal border of the depression (Figs 6D, 7D). Second exopodal segment (finger) elongate, curved at one-third its length, not acutely tapering with one medial seta proximally and two setae laterally nearly at midlength, distal part of finger with shallow depression medially.

## Discussion

The present specimens of *Calanopia thompsoni*, collected and examined from the Red Sea, closely resemble the original description by A. Scott (1909) from the Bay of Kankamaraan, south coast of Kangeang Island although the Red Sea specimens varied in the absence of a rounded ventral protuberance on the female genital compound somite. Analyses of the shape of the urosome of *Calanopia thompsoni* that have been reported in the literature between 1909 and 2008 reveal extensive variation in the shape of genital compound somite. The protuberance showed by A. Scott (1909) in the original description was absent in specimens collected from Andaman Sea (11°35'00"N, 98°34'15"E) by Sewell (1932), from the Yellow Sea by Chen and Zhang (1965) from Sister Island, Singapore waters by Othman and Toda (2006) and from Thailand waters by Phukham (2008). Also, according to these previous descriptions and illustrations of *Calanopia thompsoni* from different areas all over the world, there is some variation in the proportions of the genital compound somite (GCS) and second urosomite (UrII), as well as the first and second exopodal segments of the female leg 5. The genital compound somite is greatly variable being as long as the second urosomite to, more usually, longer than the second urosomite. Genital compound somite (GCS)/UrII = 1.2, 1.6, 1.2, 3.6 and 1.8 as described by A. Scott (1909), Chen and Zhang (1965), (1976), Othman and Toda (2006) and Phukham (2008), respectively. The Red Sea specimens are closer to the description of A. Scott from the Indonesia-Malaysia region and Silas and Pillai from the Gulf of Mannar, Indian Ocean. On the other hand, the first exopodal segment of the female leg 5 in relation to the second exopodal segment is Re1/Re2 = 0.8, 1.1, 0.6, 0.9, 0.5 and 0.6 as illustrated by A. Scott (1909), Sewell (1932), Chen and Zhang (1965), Silas and Pillai (1976), Othman and Toda (2006) and Phukham (2008), respectively. Another variation is noticed from descriptions of Chen and Zhang (1965) from Yellow Sea specimens and Mori (1937) from Japanese waters: the second (distal) exopodal segment of the male left leg 5 is longer than in other descriptions.

Some male specimens from the Red Sea revealed that the second urosomite bears two fine spinules located ventrally, on the right side. These spines are reported only in the original description of *Calanopia parathompsoni* by Gaudy (1969), on the left side. This suggests that our specimens of *Calanopia thompsoni* and Gaudy’s *Calanopia parathompsoni* are conspecific. The author of *Calanopia parathompsoni* did not explicitly designated or deposited type specimens anywhere and the species has not been illustrated since described. *Calanopia parathompsoni* was distinguished from *Calanopia thompsoni* based mainly on: 1) asymmetry of female genital compound somite in outline (absence of Scott’s protuberance), 2) presence of two fine spinules ventrally on the left side of male second urosomite, and 3) presence of a medial small spine swollen at base on the first segment of male right leg 5. Based on the examination of many specimens of *Calanopia thompsoni* from the Red Sea, we consider these differences variability within one species since the structure of leg 5 in both sexes is very similar as are the two fine spines, detected on the ventral right side of the second urosomite. Such variability is common in members of family Pontellidae (e.g., El-Sherbiny and Ueda 2008a, 2010, El-Sherbiny 2009, Jeong et al. 2009, Hirabayashi and Ohtsuka 2014). In conclusion, we are unable to find any reliable characters distinguishing *Calanopia thompsoni* and *Calanopia parathompsoni* and suggest *Calanopia parathompsoni* is a junior synonym of *Calanopia thompsoni*.

We note that the diversity of Red Sea pontellid copepods is remarkably low, given that the Indian Ocean is the origin of the Red Sea plankton. Silas and Pillai (1973) recorded 71 species of pontellid copepods from the Indian Ocean compared to 15 species from the Red Sea (*Calanopia* - 4 species, *Labidocera* - 5 species, *Pontella* - 3 species, *Pontellina* - 1 species and *Pontellopsis* - 2 species) (Razouls et al. 2015). This low number of recorded pontellid species in the Red Sea may be explained by the characteristic neustonic nature of pontellid genera (Mauchline 1998), inappropriate sampling methods or limited sampling effort in space and time. To be certain that we understand the pontellid diversity of the Red Sea, we recommend greater sampling effort.

### Key to species of *Calanopia*

**Females**

**Table:** 

1	Leg 5 exopod 1-segmented	**2**
–	Leg 5 exopod 2- segmented	**7**
2	Exopod of leg 5 with 4 spines	**3**
–	Exopod of leg 5 with 2 or 3 spines	**5**
3	Exopod of leg 5 with 3 small spines and 1 long spine	**4**
–	Exopod of leg 5 with 4 small finger like spines	***Calanopia metu***
4	Exopodal segment of leg 5 with 3 subequal small lateral spines and 1 long medial spine (longer than segment itself)	***Calanopia aurivilli***
–	Exopodal segment of leg 5 with 2 subequal lateral spines and 1 terminal long spine (nearly as long as segment)	***Calanopia americana***
5	Exopodal segment of leg 5 with 3 spines	**6**
–	Exopodal segment of leg 5 with 2 spines (lateral very short and long terminal one)	***Calanopia levantina***
6	Exopodal segment of leg 5 with 2 small lateral spines and one long medial spine (longer than segment itself)	***Calanopia minor***
–	Exopodal segment of leg 5 with 2 small lateral spines and one medial spine (smaller than segment itself)	***Calanopia kideysi***
7	Cephalic lateral hooks absent	**8**
–	Cephalic lateral hooks present	**13**
8	Leg 5 symmetrical	**9**
–	Leg 5 asymmetrical, left one longer	***Calanopia elliptica***
9	Caudal rami symmetrical	**10**
–	Caudal rami asymmetrical, right ramus much longer than left, expanded posteriorly	***Calanopia asymmetrica***
10	Second exopodal segment of leg 5 longer than first one	**11**
–	Second exopodal segment of leg 5 shorter than first one	***Calanopia herdmani***
11	First exopodal segment of leg 5 with 2 spines distally	**12**
–	First exopodal segment of leg 5 with 1 acuminate spine distally and its length nearly as long as second exopodal segment	***Calanopia sarsi***
12	Genital compound somite with ventral spines	***Calanopia media***
–	Genital compound somite without such ventral spines	***Calanopia biloba***
13	Genital compound somite longer than second urosomite	**14**
–	Genital compound somite nearly as long as second urosomite	**15**
14	Caudal rami asymmetrical, left one longer than right; second exopodal segment of leg 5 nearly as long as first one	***Calanopia australica***
–	Caudal rami slightly asymmetrical, second exopodal segment of leg 5 shorter than first one	***Calanopia seymouri***
15	Caudal rami asymmetrical, right one with more concave medial margin; second exopodal segment of leg 5 longer than first one	***Calanopia sewelli***
–	Caudal rami symmetrical; second exopodal segment of leg 5 slightly shorter than first one	***Calanopia thompsoni***

**Males (*Calanopia kideysi* and *Calanopia metu* are not included in this key since there are no descriptions for adult males)**

**Table:** 

1	Left leg 5 longer than right one; basis of left leg 5 swollen proximally	**2**
–	Left leg 5 shorter than right one; basis of left leg 5 not swollen proximally	**5**
2	Second exopodal segment of right leg 5 nearly two-fifth length of first exopodal segment; coxa of right leg 5 about or less than 1.4 times as long as basis	**3**
–	Second exopodal segment of right leg 5 nearly two-third length of first exopodal segment; coxa of right leg 5 about 1.7 times as long as basis	**4**
3	Basis of left leg 5 swollen proximally and produced into a small curved tooth	***Calanopia minor***
–	Basis of left leg 5 swollen proximally without any spines or processes	***Calanopia aurivilli***
4	Basis of left leg 5 swollen proximally and produced into a prominent tooth-like process; second exopodal segment long, second exopodal segment of leg 5 with a deep incision at the base of the thumb; third exopodal segment of right leg 5 with a distinct medial process	***Calanopia americana***
–	Basis of left leg 5 swollen proximally and produced into a small spine; second exopodal segment of leg 5 short; third exopodal segment of right leg 5 without medial process	***Calanopia levantina***
5	Prosomal posterolateral corner symmetrical	**6**
–	Prosomal posterolateral corners asymmetrical (right one wider and longer than left one and distinctly notched on its margin)	***Calanopia sarsi***
6	Cephalic lateral hooks absent	**7**
–	Cephalic lateral hooks present	**11**
7	Second urosomite symmetrical and without any processes	**8**
–	Second urosomite asymmetrical with one or two processes on right side	**10**
8	Second exopodal segment of right leg longer than first one, curved at one-third its length; basis of left leg 5 shorter than first exopodal segment	**9**
–	Second exopodal segment of right leg 5 shorter than first one, curved at mid-length with 1 short and 1 long setae; basis of left leg 5 longer than first exopodal segment	***Calanopia media***
9	First exopodal segment of right leg 5 subequal to basis, and 4.5 times as long as wide; second exopodal segment of left leg 5 with 2 lateral spines	***Calanopia asymmetrica***
–	First exopodal segment of right leg 5 shorter than basis, and 3.4 times as long as wide; second exopodal segment of left leg 5 with 1 lateral spine	***Calanopia herdmani***
10	Second urosomite with 2 processes postero-laterally on right side; left leg relatively short not reaching distal end of first exopodal segment of right leg	***Calanopia biloba***
–	Second urosomite with one acuminate-tip spinose process postero-laterally on right side; left leg relatively long reaching beyond distal end of first exopodal segment of right leg	***Calanopia elliptica***
11	Caudal rami symmetrical and divergent posteriorly; second exopodal segment of left leg nearly as long as first one	**12**
–	Caudal rami symmetrical and not divergent posteriorly; second exopodal segment of left leg 5 shorter than first one	**13**
12	First exopodal segment of right leg 5 (chela) without thump, medial margin of the chela with a group of needle-like spines and 2 smoothly curved processes; second exopodal segment of left leg 5 with short terminal spine	***Calanopia australica***
–	First exopodal segment of right leg 5 (chela) with a sharp thumb and a small seta on its lateral margin, medial margin of the chela smooth and without any processes; second exopodal segment of left leg 5 with long terminal spine	***Calanopia sewelli***
13	First exopodal segment of right leg 5 with 2 smoothly curved protuberances medially and 1 long seta proximally	***Calanopia seymouri***
–	First exopodal segment of right leg 5 with elongate, distomedial depression with 1 short seta on proximal border of depression	***Calanopia thompsoni***

## Supplementary Material

XML Treatment for Calanopia thompsoni
